# Extraction of Volatile Oil from Aromatic Plants with Supercritical Carbon Dioxide: Experiments and Modeling

**DOI:** 10.3390/molecules170910550

**Published:** 2012-09-05

**Authors:** Jose P. Coelho, Ana F. Cristino, Patrícia G. Matos, Amélia P. Rauter, Beatriz P. Nobre, Rui L. Mendes, João G. Barroso, Ana Mainar, Jose S. Urieta, João M. N. A. Fareleira, Helena Sovová, António F. Palavra

**Affiliations:** 1 Centro de Investigação de Engenharia Química e Biotecnologia, Instituto Superior de Engenharia de Lisboa, Rua Conselheiro Emídio Navarro, 1, Lisboa 1959-007, Portugal; 2 Centro de Química Estrutural, Instituto Superior Técnico, Av. Rovisco Pais, 1, Lisboa 1049-001, Portugal; 3 UL, Faculdade de Ciências de Lisboa, CCMM, Centro de Ciências Moleculares e Materiais, C8, and CQB, Centro de Química e Bioquímica, C8, and DBV, Centro de Biotecnologia Vegetal, C2, Campo Grande, Lisboa 1749-016, Portugal; 4 LNEG, Unidade de Bioengenharia (BE), Est. do Paço Lumiar 22, Lisboa 1649-033, Portugal; 5 GATHERS, Departamiento de Química Fisica, Pedro Cerbuna 12, Zaragoza 50009, Spain; 6 Institute of Chemical Process Fundamentals of the ASCR, v.v.i., Rozvojova 135, Prague 16502, Czech Republic

**Keywords:** essential oils, volatile oils, supercritical fluids, modeling, extraction

## Abstract

An overview of the studies carried out in our laboratories on supercritical fluid extraction (SFE) of volatile oils from seven aromatic plants: pennyroyal (*Mentha pulegium* L.), fennel seeds (*Foeniculum vulgare* Mill.), coriander (*Coriandrum sativum* L.), savory (*Satureja fruticosa Béguinot*), winter savory (*Satureja montana* L.), cotton lavender (*Santolina chamaecyparisus*) and thyme (*Thymus vulgaris*), is presented. A flow apparatus with a 1 L extractor and two 0.27 L separators was built to perform studies at temperatures ranging from 298 to 353 K and pressures up to 30.0 MPa. The best compromise between yield and composition compared with hydrodistillation (HD) was achieved selecting the optimum experimental conditions of extraction and fractionation. The major differences between HD and SFE oils is the presence of a small percentage of cuticular waxes and the relative amount of thymoquinone, an oxygenated monoterpene with important biological properties, which is present in the oils from thyme and winter savory. On the other hand, the modeling of our data on supercritical extraction of volatile oil from pennyroyal is discussed using Sovová’s models. These models have been applied successfully to the other volatile oil extractions. Furthermore, other experimental studies involving supercritical CO_2_ carried out in our laboratories are also mentioned.

## 1. Introduction

According to the European Pharmacopeia [1] the essential oil is the extract from aromatic plants obtained mainly by distillation processes, like hydrodistillation (HD) and steam distillation (SD). Generally, the essential oils of plants comprise monoterpenes and sesquiterpenes, plus their oxygenated derivatives, mainly alcohols, aldehydes and ketones, which are important in the food, cosmetic and pharmaceutical industries. 

These traditional extraction techniques can affect the quality of essential oil, since the degradation of thermolabile compounds, as well as the hydrolysis and hydrosolubilization of some compounds can occur. In fact, the flavor and fragrance profile of several essential oils isolated through these traditional techniques can be changed using them. To overcome these limitations a new isolation technique, supercritical fluid extraction (SFE), which is based on the solvating power of supercritical fluids, has been used in recent years as an alternative [2].

The most used supercritical fluid is CO_2_, because it is safe, low cost, allows supercritical operations at relative low pressures and near room temperatures and the extract is solvent free. SFE with CO_2_ is an environmental friendly technique, very suitable to obtain different plants extracts, because manipulations of the process parameters, such as temperature and pressure of the supercritical fluid, can change its solvent power. However, the most serious drawback of SFE, when compared with traditional atmospheric pressure extraction techniques, is the higher initial investment cost of the equipment.

In 1970 Zosel carried out at the Max Plank Institute the supercritical extraction of caffeine from coffee beans, which is considered the first technological application of this separation technique [3]. Since then a significant number of supercritical extraction studies have been published [4,5,6,7,8,9,10,11,12].

In recent years an experimental program using this new separation technique has been developed at the Laboratory of Experimental Thermodynamics (IST-Portugal) involving several aromatic plants. The oil obtained by supercritical extraction is actually designated as volatile oil to differentiate it from the essential oil, which by definition is mainly produced by SD and HD. A flow apparatus, with an extractor of 1 L and two separators of 0.27 L was built [13,14]. The best extraction conditions are selected by an experimental study along ranges of pressure and temperature, usually from 8.0 to 10.0 MPa and 313 to 323 K, respectively. Under these experimental conditions all the compounds of the volatile oil are largely soluble in supercritical CO_2_. Despite their different solubility in supercritical CO_2_ and location in aromatic plants, cuticular waxes are also coextracted. A fractionation step, after the extraction, provides, as suggested by Reverchon [15,16], the precipitation of the waxes in the first separator and the pure oil fraction in the second one.

Supercritical extraction studies of volatile oils from aromatic plants, namely, pennyroyal (*Mentha pulegium* L.) [14], fennel seeds (*Foeniculum vulgare* Mill.) [17], coriander (*Coriandrum sativum* L.) [18], savory (S*atureja fruticosa* Béguinot) [19], winter savory (*Satureja montana* L.) [20], cotton lavender (*Santolina chamaecyparisus*) [21] and thyme (*Thymus vulgaris*) [22], have been carried out. 

The SFE studies were performed under different conditions of pressure (8.0 and 10.0 MPa), temperature (313 and 323 K), mean particle size (0.3 to 0.8 mm) and CO_2_ flow rate (0.3 to 1.3 kg/h). The best compromise between yield and composition (compared with hydrodistillation) was thus obtained for the different plants species involved in the work.

The modeling of the experimental data of supercritical extraction of volatile oils from aromatic plants is very important, since it can be used as a tool for the design, improvement and scale up of this process from laboratory to pilot and industrial scale.

The most successful models in this scientific area describe the experimental process by using differential mass balances for the fluid and solid phases, like those proposed by Reverchon [23,24] and Sovová [25,26].

The first of these models [23] was applied to describe our data on pennyroyal [24] and other aromatic plants [27]. The models proposed by Sovová [25,26] were also applied to describe our data of the extraction of volatile oils from aromatic plants [27], but not pennyroyal. 

The aim of this work is to give an overview of our experimental work on supercritical extraction of volatile oils with several aromatic plants. On the other hand, the modeling of our data on supercritical fluid extraction of the volatile oil from one of the aromatic plants, pennyroyal, is discussed, in terms of the Sovová models. Furthermore, other experimental studies involving supercritical CO_2_ carried out in our laboratory are also mentioned.

## 2. Mathematical Modeling

Although a great number of experimental studies on the supercritical extraction of compounds from aromatic plants have been carried out, only a few of them have included the mathematical modeling of the results, using systematic and consistent techniques. 

The rate of supercritical extraction and the extract composition depend on many parameters, starting with the location of the extracted substance in the plant, and any mechanical and/or chemical pre-treatment of the material. Other parameters include the extractor geometry, operating conditions such as pressure, temperature and flow rate, and the conditions in the separators where the extracted substances are precipitated from the solutions. Models based on the differential mass balances for extracted compounds must include a description of the effects of these parameters on the extraction rate and yield, and enable us to design extraction experiments, to evaluate the experimental data measured under different conditions and ultimately to optimize the extraction process.

In a previous work a systematic study was developed aiming to compare several models applied to supercritical extraction of oils from aromatic plants [27]. Two models were applied, both based on mass balance equations for the fluid and solid phases. Both models assume plug flow of the solvent in the extractor, and both models distinguish between the initial quicker extraction of easily accessible extract from open cells near the particle surface, when almost saturated solution flows from the extractor, and the following slow extraction from intact cells, controlled by diffusion of extract through the low permeable cell walls.

The bed of particles in the extractor is characterized by bed void fraction, ε, content of extractable substances in the plant, *q_0_* (kg·kg^−1^), density of the plant, *ρ_s_* (kg·m^−3^), and mean size of the plant particles, *d_p_* (m). The experimental conditions are the feed of particles of plant, *N* (kg), the mass flow rate, *F* (kg·s^−1^), and CO_2_ density, *ρ_f_* (kg·m^−3^), which is pressure and temperature dependent. The mass transfer is characterized by volumetric mass transfer coefficients in the fluid phase, *k_f_a* (s^−1^), and in the intact cells in the particles, *k_s_a* (s^−1^), and on the initial fraction of easily accessible extract from the total amount of extract in the plant, *r*. The surface-to-volume ratio of the particles, a (m^2^·m^−3^), depends on their size and shape. The initial equilibrium concentration of extract in the fluid phase, expressed as a mass ratio, is C_0_ (kg·kg^−1^).

The first of those models was developed by Sovová [25]. The model was derived for supercritical fluid extraction of oil from seeds. The fluid phase equilibrium concentration is assumed to be equal to the solubility of extract in the solvent. Further, the fluid phase mass transfer coefficient is assumed to be by orders of magnitude larger than the mass transfer coefficient in the solid phase. The accumulation term in the mass balance for the fluid phase is neglected. The resulting dependence of extraction yield, *e*/(kg·(kg plant)^−1^), on extraction time, *t*/s, is:


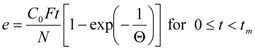
(1)


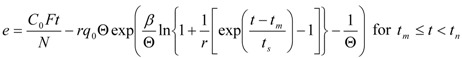
(2)


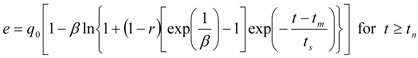
(3)

where:


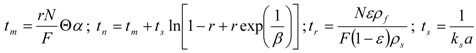
(4)


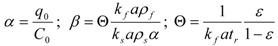
(5)

The surface-to-volume ratio was calculated according to the formula for the spheres of diameter *d_p_*:


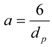
(6)

The model parameters evaluated by fitting the calculated extraction curve *e*(*t*) to experimental data are concentrations *q*_0_ and *C*_0_, mass transfer coefficients *k_f_* and *k_s_*, and the fraction of easily accessible extract *r*. As two parameters, *C*_0_ and *k_f_*, depend on one experimental quantity—the initial slope of extraction curve—they cannot be determined simultaneously from one experimental run. *C*_0_ is evaluated from a measurement for a sufficiently large residence time *t_r_*, when the slope is independent of the mass transfer coefficient. The model was applied with success [28,29] to sage oil and hiprose seed oil, respectively, as well as for pennyroyal volatile oil [24].

The second model used was also proposed by Sovová [26]. Out of the different modifications described in the original paper, the model for the extraction of substances that interact with the plant matrix was selected. A linear equilibrium relationship between the solid and fluid phases with partition coefficient *K* is assumed:


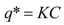
(7)

Model parameters are *q*_0_, *K*, *k_f_*, *k_s_*, and *r*. A simplified procedure of experimental data evaluation was applied. With respect to the relatively large residence times used in the experiments, the fluid phase mass transfer resistance was neglected. Two lines representing the quicker and the slower extraction periods were fitted to experimental points in a graph *e vs. t* according to the following equations: 


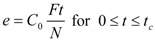
(8)


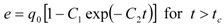
(9)

The crossing point coordinates are (*t_c_*, *C*_0_*Ft_c_*/*N*). Parameters *K*, *k_s_*, and *r* were estimated from the coefficients *C*_0_, *C*_1_ a *C*_2_. The fraction r is assumed to be equal to the fraction of extraction yield at the crossing point from the total content of extractable substances in the plant: 


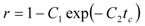
(10)

The initial concentrations at *t* = 0, when the solution starts flowing out of the extractor, were defined as follows: the concentration in the intact cells is unchanged, equal to *q*_0_. A part of the extract in open cells, however, has dissolved in the solvent and equilibrium is established between the solid and fluid phases. Combining the equilibrium relationship according to Equation (7) and mass balance in open cells and in the fluid phase we obtain for the concentration in open cells:


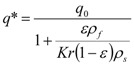
(11)

The partition coefficient is then equal to:


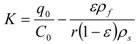
(12)

The relationship for the solid phase mass transfer coefficient, which determines the shape of the second part of the extraction curve, is derived from the model [26] as: 


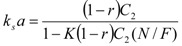
(13)

Equation (13) was used to evaluate *k_s_*. Nevertheless, the second term was under the experimental conditions negligible compared to 1 and the values of *k_s_* would be practically equal when calculated according to the simplified formula: 


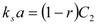
(13a)

This model has been applied with success to the extraction of oils from almond [30], fennel [31], grapes [32] and sea buckthorn [33].

In the present work we apply the models developed by Sovová [25,26] to the description of the supercritical fluid extraction of pennyroyal performed in our group [14]. Moreover, we show an evaluation of the best operating conditions for the supercritical fluid oil extraction from several plants performed in our laboratory, using those models.

## 3. Results and Discussion

Supercritical volatile oil extraction studies have been carried out for pennyroyal [14], fennel seeds [17], coriander [18], savory [19], winter savory [20], cotton lavender [21] and thyme [22]. The most efficient conditions for supercritical fluid extraction regarding yield and volatiles composition are given in Table 1.

**Table 1:** SFE conditions of the volatile oils obtain from different plants species.

Species	Common species name	Pressure (MPa)	Temperature (K)	Flow rate of CO_2_ (kg·s^−1^)	Particle Size (m)
*Mentha pulegium* L.	Pennyroyal	10.0	323	4.7 × 10^−4^	5.0 × 10^−3^
*Foeniculum vulgare ssp*. piperitum (Ucria) Coutinho	Fennel	9.0	313	3.0 × 10^−4^	6.0 × 10^−3^
*Coriandrum* *sativum* L.	Coriander	9.0	313	3.0 × 10^−4^	6.0 × 10^−3^
*Satureja montana* L.	Winter Savory	9.0	313	3.7 × 10^−4^	6.0 × 10^−3^
*Satureja fruticosa* Béguinot	Savory	9.0	313	3.0 × 10^−4^	6.0 × 10^−3^
*Santolina chamaecyparissus* L.	Cotton lavender	8.0	313	3.0 × 10^−4^	6.0 × 10^−3^
*Thymus vulgaris* L.	Thyme	9.0	313	3.7 × 10^−4^	6.0 × 10^−3^

In Table 2 and Table 3 compositions of the oils extracted from various plants by HD and SFE are presented. Concerning pennyroyal (Table 2), twenty-one components of the essential oil were identified, representing 98.1% of the total amount. The main volatile compounds were menthone (9%), menthol (1.3%), isomenthone (1%) and pulegone (80.6%), and no significant differences have been found between the compositions of the volatile and essential oils [14]. However, fennel showed a much more complex composition, where seventy components were identified in the essential oil, representing 96.5% of the total amount. The main compounds were (*E*)-anethole (42.2%), estragol (20.9%) and fenchone (16.8%), and the composition of the SFE oil is very similar to that of the essential oil obtained by hydrodistillation, although some differences were detected [17].

The SFE extract composition from coriander was compared with that obtained using hydrodistillation. Concerning both yield and composition, no major differences were observed between the two techniques used (HD and SFE) and the main components were linalool (75.9% for SFE and 67.6% for HD), γ-terpinene (5% for SFE and 6.8% for HD), camphor (3% in both cases), geranyl acetate (3.5% for SFE and 2.8% for HD), geraniol (2.9% and 2.9), α-pinene (1.5% for SFE and 2.5% for HD) and limonene (1.4% for SFE and 3.1% for HD) [18].

SFE proved to be an efficient method for extraction of volatiles from savory (Table 3) The volatiles in the SFE oil were richer in piperitenone (11.2%) and piperitenone oxide (10.7%) than those produced by hydrodistillation (9 and 6.8%, respectively), whereas pulegone (40.6%) and isomenthone (20.7%) were present in higher percentage in the essential oil from *S*. *fruticosa* then in the SFE volatiles (35.7 and 17.6%, respectively) [19].

The extracts from winter savory obtained by SFE, using the best extraction conditions, and by HD, possessed, as main compounds, carvacrol (53% in both cases), thymol (11% in both cases), *p*-cymene (12.8% for HD *vs*. 10.1% for SFE), γ-terpinene (8.9% for HD *vs*. 4.3% for SFE) and β-bisabolene (2% for HD *vs*. 2.5% for SFE). The major difference was the relative amount of thymoquinone, an oxygenated monoterpene with important biological activities. In this last aromatic plant its content can be 15-fold higher in volatile oil. The antioxidant activity of this volatile oil was studied using the DPPH and Rancimat methods [34]. The presence of carvacrol + thymol + thymoquinone in the volatile and essential oils may be responsible for their antioxidant activity.

Essential and volatile oils obtained from thyme were analysed and 52 components could be identified. The main volatile components obtained were *p-*cymene (24.4% for SFE and 28.9% for HD), γ-terpinene (2.5% for SFE and 5.1% for HD), linalool (4% for SFE and 3.1% for HD), thymol (36.3% for SFE and 41.6% for HD) and carvacrol (2.6 for SFE and 3.1% for HD). The main difference was recorded for thymoquinone (not detected in the essential oil) and carvacryl methyl ether (1.2% for HD *vs.* traces for SFE) which can explain the higher antioxidant activity, assessed by Rancimat test, of SFE volatiles when compared with HD extracted oils, as discussed in reference [22]. 

Furthermore, essential and volatile oils isolated from the flower heads of cotton lavender were obtained and the main compounds were identified. The results for both oils are presented in Table 4. These are considerably different from volatile compounds obtained from other sources in our laboratory. 1,8-Cineole was the major compound (24.8 for HD and 38.3% for SFE), followed by camphor (7.4 for HD and 10.7% for SFE), borneol (8.3 for HD and 3.8% for SFE), terpinen-4-ol (7.4 for HD and 1.9% for SFE), terpinolene (1.4 for HD and 2.1% for SFE) and isobornyl acetate (1.5 for HD and 1.2% for SFE).

In conclusion, one of the major differences between SFE and HD oils consists on the presence of small percentages of waxes. The presence of small amounts of these compounds in the oils does not seem to affect their quality, since the natural aroma was maintained. Another major difference is based on the relative amount of the biologically active thymoquinone present in the oils extracted from *Thymus vulgaris* and *Satureja Montana*. In the latter aromatic plant its content can be 15-fold higher in the volatile oil. This oxygen-containing monoterpene possesses important biological activities such as anticancer, antioxidant and antiinflamatory properties, as well as the neuroprotective effect against brain ischemia and Alzheimer’s disease [35,36,37]. Due to the pharmacological importance of S*atureja Montana* an experimental study on the extraction of antioxidants was carried out. After SFE at the best experimental conditions, shown in Table 1, all the volatile oil and waxes were extracted. Subsequently, the residual plant matrix was submitted to an extraction increasing the pressure to 25.0 MPa for 4 h. Two extracts, **E1** and **E2**, were obtained in the first and second separators which are operated, respectively, at 4.0 MPa/323 K and 2.0 MPa/293 K. 

**Table 2:** Percentage composition of the pennyroyal, fennel and coriander oils obtained by hydrodistillation and supercritical fluid extraction, in the conditions report in Table 1 [14,17,18].

Components	Pennyroyal			Fennel			Coriander	
	HD	SFE		HD	SFE		HD	SFE
α-Thujene	--	--		--	--		Tr	Tr
α-Pinene	0.3	0.2		4.6	3.5		2.5	1.5
Canfene	--	--		0.2	0.2		0.3	0.2
Sabinene	0.05	0.06		0.2	0.2		0.1	0.1
β-Pinene	0.2	0.2		1.0	0.8		0.4	0.2
Myrcene	0.09	0.1		1.4	1.4		2.8	1.0
α-Phellandrene	-	-		2.2	2.2		--	--
Octan-3-ol	0.6	0.6		--	--		--	--
α-Terpinene	--	--		--	--		0.5	0.2
p-Cymene	--	--		1.0	0.9		1.3	0.8
Limonene	0.4	0.4		3.6	3.5		3.1	1.4
*cis*-β-Ocimene	--	--		--	--		Tr	Tr
*trans*-β-Ocimene	--	--		--	--		1.7	0.4
γ-Terpinene	--	--		0.1	Tr		6.8	5.0
Terpinolene	--	--		0.6	0.6		0.9	0.5
Linalol	0.06	0.06		0.8	0.9		67.6	75.9
Fenchone	--	--		16.8	16.6		--	--
Camphor	--	--		0.6	0.4		3.0	3.1
Citronellal	--	--		--	--		0.1	0.1
Borneol	--	--		--	--		0.1	0.1
Menthone	9.0	8.5		--	--		--	--
Isomenthone	1.0	0.9		--	--		--	--
Neomenthol and neoisomenthol	2.0	2.6		--	--		--	--
Menthol	1.3	1.2		0.1	Tr		--	--
Isomenthol	0.1	0.2		--	--		--	--
Estragol	--	--		20.9	21.0		--	--
(*E*)-Anethole	--	--		42.2	42.5			
Geraniol	--	--		--	--		2.7	2.9
Pulegone	80.6	78.9		--	--		--	--
Piperitone	0.04	0.05		--	--		--	--
Isopiperitone	0.04	0.08		--	--		--	--
Neomenthyl acetate	0.7	0.6		--	--		--	--
Menthyl acetate	0.09	0.3		--	--		--	--
Isomenthyl acetate	0.05	0.06		--	--		--	--
Geranylacetate	--	--		--	--		2.8	3.5
Piperitenone	0.4	0.4		--	--			
Piperitenone oxide	--	--		0.2	0.3		--	--
β-Caryophyllene	0.4	0.5		--	--		--	--
α-Humulene	0.7	0.7		--	--		--	--
**Identified components **	98.1	96.7		96.5	95.0		97.2	97.2
**Grouped components**								
Monoterpene hydrocarbons	2.3	2.2		14.9	13.3		20.4	11.3
Oxygenated monoterpenes	95.8	94.5		81.6	81.7		76.8	85.9
Unknowns	1.9	3.3		3.5	5.0		2.8	2.8

**Table 3:** Percentage composition of the savory, winter savory and thyme volatile oils obtained by hydrodistillation and supercritical fluid extraction, in the conditions report in table. [19,20,22].

Components	Savory			Winter savory			Thyme	
	HD	SFE		HD	SFE		HD	SFE
α-Thujene	--	--		0.6	0.3		0.4	0.2
α-Pinene	0.6	0.3		0.6	0.3		0.7	0.3
Canfene	--	--					0.6	0.3
Sabinene	0.6	0.3		0.2	0.1			
Octan-3-ol		--		0.1	0.1		0.2	Tr
β-Pinene	0.4	0.4		0.8	0.6		0.7	0.9
3-Octanol							0.3	0.3
β-Myrcene	0.5	0.5		1.0	0.6		0.3	0.2
α-Phellandrene	-	--		0.2	0.2		0.1	--
Δ−3-Carene							0.1	0.2
α-Terpinene	--	--		1.7	1.2		0.7	0.5
p-Cymene	--	--		12.8	10.1		28.9	24.4
1,8-Cineole	--	--		0.5	0.4		0.3	0.3
β-Phellandrene	--	--		0.5	0.4		0.3	0.3
Limonene	1.4	1.3		0.4	0.3		0.6	0.7
*cis*-β-Ocimene	--	--		--	0.1			Tr
γ-Terpinene	--	--		8.9	4.3		5.1	2.5
Trans-sabinene hydrate	--	--		0.5	0.7		0.5	1.1
Terpinolene	--	--		0.2	0.1			
*cis*-Linalool oxide							0.1	0.2
*trans*-Linalool oxide							0.1	0.2
*cis*-Sabinene hydrate	--	--		0.1	0.2		Tr	0.3
Linalol		--		0.8	0.7		3.1	4.0
Camphor	--	--					0.8	0.9
Borneol	--	--		0.7	0.7		1.2	1.2
Terpinen-4-ol	--	--		0.7	0.4		0.8	0.7
*cis* -Dihydrocarvone	--	--		--			0.3	0.3
Menthone	0.5	0.3		--				--
Isomenthone	20.7	17.6		--				--
α-Terpineol	0.2	0.2		0.2	0.2		0.2	0.2
Carvone	--	--		--	0.2			
Thymoquinone	--	--		0.2	2.9		Tr	6.2
Pulegone	40.6	35.7		--	--			--
Thymyl methyl ether							0.2	Tr
Carvacrol methyl ether	--	--		0.1	--		1.2	Tr
Geraniol							0.1	0.2
Thymyl formate							0.3	Tr
Thymol	--	--		11.0	10.9		41.6	36.3
Carvacrol	--	--		52.2	52.7		3.1	2.6
β-Bourbonene	--	--		0.1	0.1		0.1	0.2
4-Ethyl-2-methoxy-6-methylphenol							1.2	1.6
Piperitone oxide + Piperitone	10.5	9.8						
Piperitenone	9.0	11.2		--				
Piperitenone oxide	6.8	10.7						--
β-Caryophyllene	1.2	1.2		1.3	1.5		1.2	1.6
Trans-bergamotene	--	--		0.2	0.2			
β-Copaene							0.2	2.0
α-Humulene							0.1	Tr
γ-Muurolene	--	--		0.1	0.1		0.3	0.3
Germacrene D	1.3	1.6		0.2	0.3		0.1	0.2
β-Bisabolene	--	--		2.0	2.5			
α-Muurolene							0.1	0.2
*trans-*Calamenene							0.3	0.3
Δ-Cadinene	--	--		0.2	0.2		0.4	0.2
Thymohydroquinone	--	--		0.4	0.5			--
β-Caryophyllene oxide	--	--		0.2	0.2		1.3	1.6
epi-α-Cadinol							0.1	0.3
**Identified components **	94.3	91.1		99.0	96.0		97.3	94.4
**Grouped components**								
Monoterpene hydrocarbons	3.5	2.8		27.8	20.3		39.0	31.9
Oxygenated monoterpenes	88.3	85.5		66.8	70.4		54.8	54.9
Sesquiterpene hydrocarbons	2.5	2.8		4.4	5.1		2.9	5.5
Oxygenated sesquiterpene	--	--		0.2	0.2		1.4	2.1
Unknowns	5.7	8.9		1.0	4.0		2.7	5.6

**Table 4:** Percentage composition of cotton lavender oils obtained by HD and SFE, in the conditions report in Table 1 [21].

Components	HD	SFE
α-Thujene	--	t
α-Pinene	0.1	0.2
Camphene	0.8	0.9
β-Pinene	1.3	1.6
1,8-Dehydrocineole	0.2	t
2-Pentylfuran	0.3	t
Yomogi alcohol	0.3	0.7
α-Terpinene	2.4	1.9
*p*-Cymene	2.0	2.1
1,8-Cineole	24.8	38.3
Artemisia ketone	0.3	t
*trans*-Sabinene hydrate	3.1	1.7
Terpinolene	1.4	2.1
*cis*-Sabinene hydrate	0.8	0.6
*n*-Nonanal	0.4	0.6
Linalool	0.2	0.3
Isopentyl isovalerate	0.2	t
α-Campholenal	0.3	0.7
*trans*-*p*-Menthen-1-ol	0.7	--
Camphor	7.4	10.7
Verbenol	0.2	0.3
Borneol	8.3	3.8
Thuj-3-en-10-al	0.8	0.8
Terpinen-4-ol	7.4	1.9
Myrtenal	0.4	0.4
α-Terpineol	0.2	t
Myrtenol	1.7	1.7
*trans*-Carveol	0.2	t
*p*-Cymen-7-ol	0.3	0.2
Isobornyl acetate	1.5	1.2
Thymol	0.1	0.2
*trans*-Ascaridol	t	--
Carvacrol	0.3	--
2 *trans*, 4 *trans*-Decadienal	0.2	0.4
Myrtenyl acetate	0.2	0.2
Bicycloelemene	t	0.5
α-Aromandendrene	1.3	1.4
Germacrene-D	2.7	2.9
Bicyclogermacrene	1.4	1.0
γ-Cadinene	0.2	0.2
δ-Cadinene	0.3	t
α-Calacorene	t	t
Spathulenol	3.1	0.6
β-Caryophyllene oxide	1.0	t
Globulol	0.1	t
Viridiflorol	0.1	t
Anhydro oplopanone	1.0	t
*epi*-α-Cadinol	1.1	0.2
Hexadecanoic acid	0.2	t
*n*-Heneicosane	t	0.3
*n*-Tricosane	t	--
*n*-Tetracosane	0.2	0.1
*n*-Octacosane	0.1	t
*n*-Hexatriacosane	0.1	0.3
**Identified components**	81.7	81.0
**Grouped components**		
Monoterpene hydrocarbons	8.0	8.8
Oxygen-containing monoterpenes	59.7	63.7
Sesquiterpene hydrocarbons	5.9	6.0
Oxygen-containing sesquiterpenes	6.4	0.8
Others	1.7	1.7

Table 5 shows the chemical profile of both extracts determined by HPLC-DAD. The two extracts contain the same polyphenols. However, the extract E2 is richer in antioxidants (+)-catechin, protocatechuic acid and vanillic acid and the bioactivity exhibited by these extracts can be probably explained by this chemical profile [35].

**Table 5:** Chemical profile of the supercritical extracts from *Satureja Montana* (relative percentage).

Phenolic compound	Extract, E1(%)	Extract, E2 (%)
Protocatecheuic acid	0.18	0.38
Chlorogenic acid	0.60	0.75
Gallic acid	1.35	0.14
Gentisic acid	0.10	0.03
Vanillic acid	0.21	1.03
Caffeic acid	0.46	0.21
(+) – catechin	0.25	0.67
(−) – epicatechin	0.20	0.75
Syringic acid	0.32	0.19
Ferrulic acid	0.40	0.42
Coumaric acid	0.52	0.22

Another important application of the essential oils from coriander, winter savory and thyme is their potential use as natural herbicide. In view of our preliminary results, the volatile oils from thyme appear as a promising alternative to the synthetic herbicides, as its extract has shown the least injurious effect on crop species. In contrast, the essential oil from winter savory, affected both crop and weeds and, for this reason, it looks more appropriate for uncultivated fields [38].

The modeling of our data on supercritical fluid extraction of the volatile oil from the above mentioned species has been performed [27] using the two previously described models proposed by Sovová [25,26]. A more detailed study will be presented to pennyroyal considering condition of pressure and temperature of 10.0 MPa and 323 K, respectively, flow rate of 4.7 × 10^−4^ kg·s^−1^ and different particle sizes of 0.3, 0.5 and 0.75 mm.

**Figure 1:**
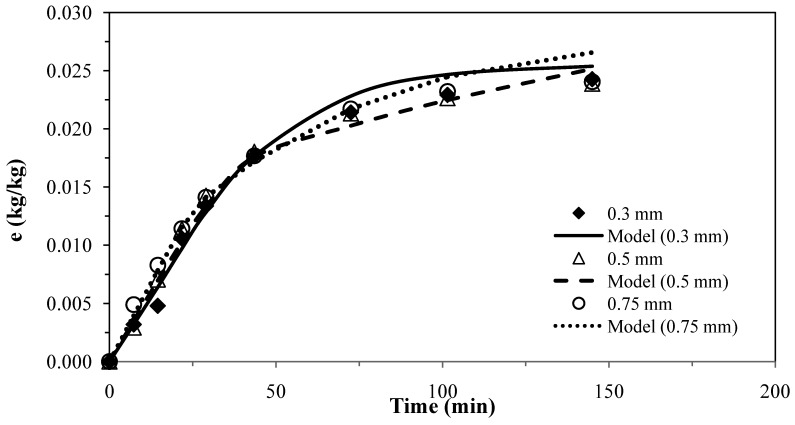
Pennyroyal essential oil yield for different mean particle size and and CO_2_ flow rate of 4.7 × 10^−4^ kg·s^−1^. Continuous curves from model, k_s_ = 0.89 × 10^–8^ m·s^−1^ and k_f_= 1.95 × 10^−6^ m·s^−1^.

The results for the first model [25] for pennyroyal are shown in Figure 1. The parameters obtained for the different particle sizes and the absolute average deviations are shown in Table 6. 

**Figure 2:**
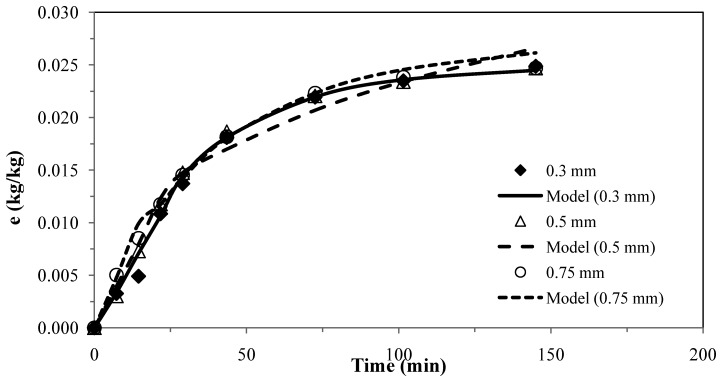
Pennyroyal essential oil yield for different mean particle size and and CO_2_ flow rate of 4.7 × 10^−4^ kg·s^−1^. Continuous curves from model, k_s_ = 1.50 × 10^–8^ m·s^−1^.

The results for the second model [26] for pennyroyal are presented in Figure 2. The parameter obtained for different particle sizes and the absolute average deviations are shown in Table 6.

**Table 6:** Mass transfer coefficients for pennyroyal volatile oil extraction considering pressure and temperature of 10.0 MPa and 323 K, respectively, flow rate of 4.7 × 10^−4^ kg·s^−1^.

Particle size (mm)	Sovová Model [25] k_s_× 10^8^ (m·s^−1^)/k_f_× 10^6^ (m·s^−1^)	Sovová Model [26] k_s_× 10^8^ (m·s^−^^1^)
0.3	3.39/0.246	4.93
	(8.5%)	(5%)
0.5	8.94/1.95	1.50
	(6.4%)	(8.4%)
0.75	4.60/8.29	8.58
	(4.9%)	(3%)

The experimental results obtained with three different particles sizes using two or only one adjustable parameter, provided a fairly good agreement between the model curves and the experimental data, as shown in Figure 1 and Figure 2, which evidence the capability of these models.

These two models were applied for the best conditions of SFE for each plant shown in Table 1. Table 7 presents the AAD (%) and the mass transfer parameters for the different matrices, showing that the two models do not present significant overall differences for describing the supercritical extraction of volatile oils from the plants. The extraction curves measured for different particle sizes in Figure 1 and Figure 2 seem to overlap as if there is no effect of particle size. This is in accordance with the fact that the essential oil is extracted from glands on the surface of pennyroyal leaves and therefore any milling of the leaves does not make the oil more accessible.

## 4. Others Supercritical Fluid Extraction Projects

Others projects have been also developed in our laboratories in the following areas.

### 4.1. Supercritical Fluid Extraction of Biological Compounds from Microalgae

More than several thousand species of microalgae are supposed to exist in the World, although only less than a hundred have been well studied [39]. Due to their photosynthetic capability several microalgae have been cultivated in open ponds at the Unit of Bioenergy (UB) of LNEG (Portugal). This method is the most appropriate for species such as *Chlorella vulgaris*, *Haematococcus pluvialis*, *Dunaliella salina*, *Botryococcues braunii* and *Arthrospira* (*Spirulina maxima*). Supercritical fluid extraction studies of bioactive compounds from these microalgae have been mainly carried out at this Unit using a semi-continuous flow apparatus built at UB of LNEG and described in detail by Mendes *et al.* [40].

**Table 7:** Parameters of initial conditions for fluid (C_o_) and solid (q_o_) phases, and mass transfer coefficients, (k_s_) of the different plants species involved in the studies with supercritical fluid extractions, in the conditions reported in Table 1 AAD% is shown within parenthesis.

Species	C_o_ (kg/kg) × 10^3^	q_o_ (kg/kg) × 10^3^	Model Sovová [25] k_s_× 10^8^ (m·s^−1^)/k_f_× 10^6^ (m·s^−1^)	Model Sovová [26] k_s_× 10^8^ (m·s^−^^1^)
*Mentha pulegium* L.	1.40	34.37	0.89/ 1.95 (6.4)	1.50 (8.4)
*Foeniculum vulgare ssp*. piperitum (Ucria) Coutinho	1.70	32.62	3.05/4.50 (1.0)	3.91 (2.9)
*Coriandrum* *sativum* L.	1.05	4.43	1.30/1.18 (3.8)	1.38 (1.5)
*Satureja montana* L.	2.10	32.00	1.31/1.95(4.9)	1.85 (3.8)
*Satureja fruticosa* Béguinot	1.10	17.30	0.42/0.88(1.6)	0.31 (5.6)
*Santolina chamaecyparissus* L.	0.70	2.50	8.43/17.62(17.0)	14.88 (6.7)
*Thymus vulgaris* L.	1.00	11.27	1.26/0.60(6.5)	1.53 (4.1)

Several supercritical fluid extraction studies of bioactive compounds from microalgae, such as hydrocarbons from *Botryococcus braunii* [40,41,42,43], lipids and carotenoids (*i.e.*, astaxanthin, cantaxanthin) from *Chlorella vulgaris* [40,42,43,44,45], β-carotene from *Dunaliella salina* [42], carotenoids (e.g., astaxanthin, cantaxanthin and β-carotene) from *Haematoccus pluvialis* [46] and lipids and fatty acids (γ-linolenic acid) from *Artrosphira Spirulina maxima* [42,47,48], have been carried out. Moreover, modeling studies of the supercritical fluid extraction experiments, using mass transfer models, have been performed for the extraction of hydrocarbons from *Botryococcus braunii* [49,50], astaxanthin and cantaxanthin from *Chlorella vulgaris* [49,50,51], and fatty acids from *Artrosphira Spirulina maxima* [47,52,53].

### 4.2. Solubility of Carotenoids in Supercritical Fluids

Solubility data are very important in several fields of supercritical technology, such as chromatography, extraction, and crystallization. The semi-continuous flow apparatus built at LNEG, with an extractor of 32 mL, was used to carry out solubility studies of biological compounds in the temperature range from 308 to 333 K and pressures up to 40.0 MPa. This apparatus was tested through solubility measurements of naphthalene [40].

Studies [54,55] have shown that for compounds with low solubility in the supercritical fluid the influence of the purity of the starting material could be an important concern. In fact, the presence of impurities or degradation compounds in the samples can affect the obtained solubility values in supercritical fluids. Therefore, it is necessary to eliminate these interfering compounds in order to obtain reliable solubility data and supercritical fluids have been shown to be a good tool for this purpose [54].

Several solubility studies of biological compounds in supercritical fluids have been performed, such as β-carotene in supercritical CO_2_ and ethane [54], bixin and bixin + β-carotene in supercritical CO_2_ [55]. On the other hand, the solubility of β-carotene was also measured in two near-critical solvent mixtures (ethane + propane) [56], as well as that of *cis* and *trans* β-carotene isomers, obtained from the microalga *Dunaliella salina* in supercritical CO_2_ [57,58].

Furthermore, the modeling of the above mentioned solubility measurements using the density-based empirical model, proposed by Chrastil [56,58], as well as the Peng-Robinson equation of state [54,55] was also carried out.

### 4.3. Micronization of Pharmaceutical Compounds Using Supercritical Fluids

Active pharmaceutical ingredients (APIs) are used in pharmaceutical products in the form of dry powders, liquid and semi-solid dispersions, which can range from nanocolloids to microparticles, depending on the dosage and the administration usage. The size and the shape of the API particle will have a considerable impact in the physical properties, production processes and quality attributes of the final product. Moreover, in cream and ointment formulations for topical and ophthalmic applications, the particle size and particle size distribution can play an important role in the final product, since large particles can be abrasive for the macula of the eyes. Therefore, the production of micro- and nanoparticles is an important subject for the pharmaceutical industry, as well as the process of micronization. Supercritical anti-solvent micronization (SAS) studies of pharmaceutical compounds, such as minocycline hydrochloride [59] and *trans*-β-carotene [60] have been carried out using a SAS apparatus. More recently, similar studies are being completed on fusidic acid and sodium fusidate.

A new mathematical model was also implemented to understand the process of supercritical antisolvent micronization of minocycline hydrochloride [61]. This model gathered mass transfer, jet hydrodynamics and phase equilibrium data with the buoyancy effects on fluids dynamics. The resolution of the model by computer fluid dynamics (CFD) allowed discussing the effects of the concentration and the flow-rate of organic solution in the particle size distribution.

## 5. Experimental

### 5.1. Supercritical Fluid Extraction Apparatus

A flow apparatus was built at Laboratory of Experimental Thermodynamics which allows carrying out supercritical extraction studies in the temperature range of 298 to 353 K and pressures up to 30.0 MPa [13,14]. Liquid CO_2_ from a cylinder was compressed with high pressure pump. Before flowing through an extraction vessel (1 L), the CO_2_ is heated in a heater exchanger. A back-pressure regulator is used to control the pressure, which is measured with a Bourdon type manometer. A pre-set temperature in the extraction vessel is reached with the aid of a water jacket. The supercritical CO_2_ after the extraction is expanded into two separators (0.27 L), being the corresponding pressure measured with two manometers. On the other hand, the temperature of both separators is controlled through two thermostatic baths. Furthermore, the flow rate of CO_2 _was measured with a rotameter and the total volume determined with a dry test meter.

### 5.2. Extraction

*SFE*: For each run of supercritical CO_2_ extraction, 70–120 g, of plant material was used to fill the extractor. Extraction conditions were: CO_2_ flow rates of 0.71, 1.32 and 1.64 kg/h and pressures of 8.0 to 10.0 MPa at the temperature of 313–323 K. For the fractionation steps, a pressure of 8.0 MPa and a temperature of 273 K, in the first separator, and a pressure of 2.0 MPa and a temperature of 265 K, in the second one, were considered suitable to the experiments. The amount of supercritical volatile oil, obtained in the second separator, was determined gravimetrically (w/w) as a function of the time. In the first separator, only waxes were collected and quantified at the end of the assay and were not taken in account to the present work. 

*HD*: Hydrodistillation was carried out, for 4 h, in a Clevenger-type apparatus, using 40 g of the plant material with the same average particle size used in the supercritical extraction conditions.

### 5.3. Analytical Methods

#### 5.3.1. Gas Chromatography

Quantitative analysis was performed in a Hewlett-Packard 5890 gas chromatograph, using a flame ionization detector (FID) and a fused-silica DB-5 capillary column (J&W Scientific Inc, Folsom, CA, USA; 30 m × 0.25 mm i.d., film thickness 0.25 μm). The percentage composition of the oils was computed by the normalization method from the GC peak areas without using response factors.

#### 5.3.2. Gas Chromatography-Mass Spectrometry.

A Perkin Elmer Autosystem XL gas chromatograph equipped with DB-1 fused-silica column (30 m × 0.25 mm i.d., film thickness 0.25 μm; J&W Scientific Inc.), interfaced with a Perkin Elmer Turbomass mass spectrometer (software version 4.1) was used to perform the GC-MS analyses. The identification of the components was assigned by comparison of their retention indices, relative to C_9_–C_16_ *n*-alkane indices, and to GC-MS spectra from a home-made library, based on the analysis of commercially available standards and laboratory synthesized components. 

## 6. Conclusions

This paper presents an overview of our work on supercritical fluid extraction of volatile oils from aromatic plants. Particularly interesting are the recent studies to obtain nonvolatile extracts from winter savory using CO_2_ at 25.0 MPa. The potential of these extracts for treatment of human degenerative dementias such as Alzheimer’s disease, was mentioned.

The antioxidant activity of some of the SFE extracts can be rationalized in terms of their antioxidant components, that render them unique candidates for functional foods for the prevention and treatment of diabetes, cancer and cardiovascular diseases, amongst others where the oxidative stress plays also an important role. The paper also presents a successful modeling study of the extraction of volatile oils from pennyroyal, using two models developed by Sovová.

This project is a multidisciplinary one involving several national and international collaborations. The know-how acquired during its implementation allowed the development of other collaborative projects in the area of supercritical fluids, such as supercritical extraction of bioactive compounds from microalgae, solubility of carotenoids in supercritical fluids and micronization of pharmaceutical compounds using supercritical fluids. Furthermore, recently a new collaborative project in the scientific area of catalytic oxidation of hydrocarbons using supercritical CO_2_ as reaction medium has been developed [62].

## References

[1] Council of Europe (2007). Council of Europe- COE- European Directorate for Quality of Medicines, European Pharmacopeia.

[2] Bruno T.J., Castro C.A.N., Hamel J.F.P., Palavra A.M.F., Kennedy J.F., Cabral J.M.S. (1993). Process for Biological Materials.

[3] Zosel K. (1978). Separation with supercritical gases: Practical applications. Angew. Chem..

[4] Reverchon E. (1997). Supercritical fluid extraction and fractionation of essential oils and related products. J. Supercrit. Fluid..

[5] Meireles M.A.A. (2003). Supercritical extraction from solid: Process design data (2001–2003). Curr. Opin. Solid State Mat. Sci..

[6] Reverchon E., De Marco I. (2006). Supercritical fluid extraction and fractionation of natural matter. J. Supercrit. Fluid..

[7] Pourmortazavi S.M., Hajimirsadeghi S.S. (2007). Supercritical fluid extraction in plant essential and volatile oil analysis. J. Chromatogr. A.

[8] Munshi P., Bhaduri S. (2009). Supercritical CO_2_: A twenty-first century solvent for the chemical industry. Curr. Sci..

[9] Herrero M., Mendiola J.A., Cifuentes A., Ibánez E. (2010). Supercritical fluid extraction: Recent advances and applications. J. Chromatogr. A.

[10] Gañán N., Brignole E.A. (2011). Fractionation of essential oils with biocidal activity using supercritical CO_2_—Experiments and modeling. J. Supercrit. Fluid..

[11] Sovová H. (2012). Modelling the supercritical fluid extraction of essential oils from plant material. J. Chromatogr. A.

[12] Fornari T., Vicente G., Vázquez E., García-Risco M.R., Reglero G. (2012). Isolation of essential oil from different plants and herbs by supercritical fluid extraction. J. Chromatogr. A.

[13] Reis-Vasco E.M.C., Coelho J.A.P., Palavra A.M.F. Extraction of Pennyroyal Oil (*Mentha pulegium* L.) with Supercritical CO_2_. Proceedings of Fourth Italian Conference on Supercritical Fluids and their Applications.

[14] Reis-Vasco E.M.C., Coelho J.P., Palavra A.F. (1999). Comparison of pennyroyal oils obtained by supercritical CO_2_ extraction and hydrodistillation. Flavour Frag. J..

[15] Reverchon E. (1992). Fractional separation of SCF extracts from majoram leaves: Mass transfer and optimization. J. Supercrit. Fluid..

[16] Reverchon E., Senatore F. (1992). Isolation of rosemary oil: Comparison between hydrodistillation and supercritical CO_2_ extraction. Flavour Frag. J..

[17] Coelho J.A.P., Pereira A.P., Mendes R.L., Palavra A.M.F. (2003). Supercritical carbon dioxide extraction of *Foeniculum vulgare* volatile oil. Flavour Frag. J..

[18] Grosso C., Ferraro V., Figueiredo A.C., Barroso J.G., Coelho J.A., Palavra A.M. (2008). Supercritical carbon dioxide extraction of volatile oil from Italian coriander seeds. Food Chem..

[19] Coelho J.A.P., Grosso C., Pereira A.P., Burillo J., Urieta J.S., Figueiredo A.C., Barroso J.G., Mendes R.L., Palavra A.M.F. (2007). Supercritical carbon dioxide extraction of volatiles from *Satureja fruticosa* Béguinot. Flavour Frag. J..

[20] Grosso C., Figueiredo A.C., Burillo J., Mainar A.M., Urieta J.S., Barroso J.G., Coelho J.A., Palavra A.M.F. (2009). Enrichment of the thymoquinone content in volatile oil from *Satureja montana* using supercritical fluid extraction. J. Sep. Sci..

[21] Grosso C., Figueiredo A.C., Burillo J., Mainar A.M., Urieta J.S., Barroso J.G., Coelho J.A., Palavra A.M. (2009). Supercritical fluid extraction of the volatile oil from *Santolina chamaecyparissus*. J. Sep. Sci..

[22] Grosso C., Figueiredo A.C., Burillo J., Mainar A.M., Urieta J.S., Barroso J.G., Coelho J.A., Palavra A.M. (2010). Composition and antioxidant activity of *Thymus vulgaris* volatiles: Comparison between supercritical fluid extraction and hydrodistillation. J. Sep. Sci..

[23] Reverchon E., Della Porta G., Lamberti G. (1999). Modelling of orange flower concrete fractionation by supercritical CO_2_. J. Supercrit. Fluid..

[24] Reis-Vasco E., Coelho J.A.P., Palavra A.M.F., Marrone C., Reverchon E. (2000). Mathematical modeling and simulation of pennyroyal essential oil supercritical extraction. Chem. Eng. Sci..

[25] Sovová H. (1994). Rate of the vegetable oil extraction with supercritical CO_2_—I. Modelling of extraction curves. Chem. Eng. Sci..

[26] Sovová H. (2005). Mathematical model for supercritical fluid extraction of natural products and extraction curve evaluation. J. Supercrit. Fluid..

[27] Grosso C., Coelho J.P., Pessoa F.L.P., Fareleira J.M.N.A., Barroso J.G., Urieta J.S., Palavra A.F., Sovová H. (2010). Mathematical modeling of supercritical CO_2_ extraction of volatile oils from aromatic plants. Chem. Eng. Sci..

[28] Reverchon E. (1996). Mathematical modeling of supercritical extraction of sage oil. AIChE J..

[29] Reverchon E., Kaziunas A., Marrone C. (2000). Supercritical CO_2_ extraction of hiprose seed oil: Experiments and mathematical modeling. Chem. Eng. Sci..

[30] Marrone C., Poletto M., Reverchon E., Stassi A. (1998). Almond oil extraction by supercritical CO_2_: Experiments and modeling. Chem. Eng. Sci..

[31] Reverchon E., Daghero J., Marrone C., Mattea M., Poletto M. (1999). Supercritical fractional extraction of fennel seed oil and essential oil: Experiments and mathematical modeling. Ind. Eng. Chem. Res..

[32] Sovová H., Kučera J., Jež J. (1994). Rate of the vegetable oil extraction with supercritical CO_2_—II. Extraction of grape oil. Chem. Eng. Sci..

[33] Šastová J., Jež J., Bártlová M., Sovová H. (1996). Rate of the vegetable oil extraction with supercritical CO_2_. III. Extraction from sea buckthorn. Chem. Eng. Sci..

[34] Grosso C., Oliveira A.C., Mainar A.M., Urieta J.S., Barroso J.G., Palavra A.M.F. (2009). Antioxidant activities of the supercritical and conventional of *Satureja montana* extracts. J. Food Sci..

[35] Silva F.V.M., Martins A., Neng N.R., Nogueira J.M.F., Mira D., Gaspar N., Justino J., Grosso C., Urieta J.S., Palavra A.M.F. (2009). Phytochemical profile and anticholinesterase and antimicobial activities of supercritical versus conventional extracts of *Satureja montana*. J. Agric. Food Chem..

[36] Gali-Muhtasib H., Roessner A., Schneider-Stock R. (2006). Thymoquinone: A promising anti-cancer drug from natural sources. Int. J. Biochem. Cell Biol..

[37] Jukic M., Politeo O., Maksimovic M., Milos M., Milos M. (2006). *In vitro* acetylcholinesterase inhibitory properties of thymol, carvacrol and their derivatives thymoquinone and thymohydroquinone. Phytother. Res..

[38] Grosso C., Coelho J.A., Urieta J., Palavra A.M.F., Barroso J.G. (2010). Herbicidal activity of volatiles from coriander, winter savory, cotton lavender, and thyme isolated by hydrodistillation and supercritical fluid extraction. J. Agric. Food Chem..

[39] Borowitzka M.A., Borowitzka M.A., Borowitzka L.J. (1988). Vitamins and fine chemicals from microalgae. Micro-algal Biotechnology.

[40] Mendes R.L., Coelho J.P., Fernandes H.L., Marrucho I.J., Cabral J.M.S., Novais J.M., Palavra A.F. (1995). Applications of supercritical CO_2_ extraction to microalgae and plants. J. Chem. Technol. Biotechnol..

[41] Mendes R.L., Fernandes H.L., Coelho J.A.P., Cabral J.M.S., Novais J.M. (1994). Supercritical carbon dioxide extraction of hydrocarbons from the microalga *Botryococcus braunii*. J. Appl. Phycol..

[42] Mendes R.L., Nobre B.P., Cardoso M.T., Pereira A.P., Palavra A.F. (2003). Supercritical carbon dioxide extraction of compounds with pharmaceutical importance from microalgae. Inorg. Chim. Acta.

[43] Palavra A.M.F., Coelho J.P., Barroso J.G., Rauter A.P., Fareleira J.M.N.A., Mainar A., Urieta J.S., Nobre B.P., Gouveia L., Mendes R.L. (2011). Supercritical carbon dioxide extraction of bioactive compounds from microalgae and volatile oils from aromatic plants. J. Supercrit. Fluid..

[44] Mendes R.L., Fernandes H.L., Coelho J.P., Reis E.C., Cabral J.M.S., Novais J.M., Palavra A.F. (1995). Supercritical CO_2_ extraction of carotenoids and other lipids from *Chlorella vulgaris*. Food Chem..

[45] Gouveia L., Nobre B.P., Marcelo F.M., Mrejen S., Cardoso M.T., Palavra A.F., Mendes R.L. (2007). Functional food oil coloured by pigments extracted from microalgae with supercritical CO_2_. Food Chem..

[46] Nobre B., Marcelo F., Passos R., Beirão L., Palavra A., Gouveia L., Mendes R. (2006). Supercritical carbon dioxide extraction of astaxanthin and other carotenoids from the microalga *Haematococcus pluvialis*. Eur. Food Res. Technol..

[47] Mendes R.L., Reis A.D., Pereira A.R., Cardoso M.T., Palavra A.F., Coelho J.P. (2005). Supercritical CO_2_ extraction of gamma-linolenic acid (GLA) from the cyanobacterium *Arthrospira* (*Spirulina*) *maxima*: Experiments and modeling. Chem. Eng. J..

[48] Mendes R.L., Reis A.D., Palavra A.F. (2006). Supercritical CO2 extraction of gamma-linolenic acid and other lipids from *Arthrospira* (*Spirulina*) *maxima*: Comparison with organic solvent extraction. Food Chem..

[49] Mendes R.L., Fernandes H.L., Provost M.L., Reis E.L., Cabral J.M.S., Novais J.M., Palavra A.M.F. (1996). Modelisation de L’Exctraction Supercritique de Lípides D’Algues. Proceedings of 3eme Colloque sur les Fluids Supercritiques Applications Aux Produits Naturels: Arômes, Parfoms, Plantes Médicinais.

[50] Mendes R.L., Palavra A.M.F. (1998). Supercritical CO_2 _Extraction of Compounds from Microalgae. Proceedings of Third Workshop on ChemistryEnergy and Environment.

[51] Mendes R.L., Fernandes H.L., Provost M.C., Cabral J.M.S., Novais J.M., Palavra A.M.F. Supercritical CO_2_ Extraction of Lipids from Microalgae. Proceedings of the 3rd International Symposium on Supercritical Fluid.

[52] Mendes R.L., Reis A., Fernandes H.L., Novais J.M., Palavra A.M.F. Supercritical CO_2_ Extraction of Lipids from a GLA-Rich *Arthrospira (Spirulina) maxima* Biomass. Proceedings of the Fifth Conference on Supercritical Fluids and their Applications.

[53] Pereira A.P., Coelho J.P., Nobre B.P., Reis A.D., Mendes R.L., Palavra A.M.F. Supercritical CO_2_ Extraction of GLA and Other Lipids from the Cyanobacterium *Spirulina maxima*. Proceeding of the 7th Meeting on Supercritical FluidsParticle Design-Materials and Natural Products Processing.

[54] Mendes R.L., Nobre B.P., Coelho J.P., Palavra A.F. (1999). Solubility of Beta-Carotene in Supercritical Carbon Dioxide and Ethane. J. Supercrit. Fluid..

[55] Nobre B.P., Mendes R.L., Queiros E.M., Pessoa F.P., Coelho J.P., Palavra A.F. (2009). Calculations of solubilities for systems containing multiple non-volatile solutes and supercritical carbon dioxide. Ind. Eng. Chem. Res..

[56] Nobre B.P., Palavra A.F., Mendes R.L. (2002). Solubility of Beta-Carotene in Near-Critical Mixtures of (Ethane + Propane). J. Chem. Eng. Data.

[57] Mendes R.L., Fernandes H.L., Roxo C.H., Novais J.M., Palavra A.F. Solubility of Synthetic and Natural β-carotene in Supercritical Carbon Dioxide. Proceedings of the 4th Italian Conference on Supercritical Fluids and Their Applications.

[58] Nobre B.P., Palavra A.F., Mendes R.L. Correlation of the Solubility of β-carotene in Supercritical Carbon Dioxide and Ethane with Chrastil and Reid Equations. Proceedings of the 5th Conference on Supercritical Fluids and their Applications.

[59] Tavares Cardoso M.A., Monteiro G.A., Cardoso J.P., Prazeres T.J.V., Figueiredo J.M.F., Martinho J.M.G., Cabral J.M.S., Palavra A.M.F. (2008). Supercritical antisolvent micronization of minocycline hydrochloride. J. Supercrit. Fluid..

[60] Cardoso M.A.T., Antunes S., van Keulen F., Ferreira B.S., Geraldes A., Cabral J.M.S., Palavra A.M. (2009). Supercritical antisolvent micronisation of synthetic all-*trans*-β-carotene with tetrahydrofuran as solvent and carbon dioxide as antisolvent. J. Chem. Technol. Biotechnol..

[61] Tavares Cardoso M.A., Cabral J.M.S., Palavra A.M.F., Geraldes V. (2008). CFD analysis of supercritical antisolvent (SAS) micronization of minocycline hydrochloride. J. Supercrit. Fluid..

[62] Fernandes R.R., Jamal Lasri J., Guedes da Silva M.F.C., Palavra A.M.F., Silva J.A.L., Frausto da Silva J.J.R., Pombeiro A.J.L. (2011). Oxadiazoline and Ketoimine Palladium (II) Complexes as Highly Efficient catalysts for Suzuki-Miyaura Cross-Coupling Reactions in Supercritical Carbon Dioxide. Adv. Synth. Catal..

